# Pharmacokinetics of Efmoroctocog alfa by Two-Compartment Model Highlights Hemophilia A Patients with Biphasic Decay, Long Mean Residence Time, and Beta Half-Life

**DOI:** 10.3390/jcm13174986

**Published:** 2024-08-23

**Authors:** Massimo Morfini, Flora Peyvandi, Maria Elisa Mancuso, Emanuela Marchesini, Annarita Tagliaferri, Roberta Gualtierotti, Giancarlo Castaman, Berardino Pollio, Cristina Santoro, Luisa Banov, Mariasanta Napolitano, Paola Stefania Preti, Rita Carlotta Santoro, Antonio Coppola, Silvia Linari, Elena Santagostino, Francesco Bernardi

**Affiliations:** 1 Italian Association of Hemophilia Centers (AICE), I 20120 Milan, Italy; 2 Department of Pathophysiology and Transplantation, University of Milan, Fondazione IRCSS Ca’ Granda Maggiore Hospital, “Angelo Bianchi Bonomi”, I 20120 Milan, Italy; flora.peyvandi@unimi.it (F.P.); roberta.gualtierotti@unimi.it (R.G.); 3 Center for Thrombosis and Hemorrhagic Diseases, IRCCS “ Humanitas Research Hospital”, Rozzano, I 20142 Milan, Italy; mariaelisa.mancuso@humanitas.it; 4 Hemophilia Centre, Internal and Cardiovascular Medicine, “Santa Maria della Misericordia” University Hospital, I 06100 Perugia, Italy; emanuela.marchesini@ospedale.perugia.it; 5 Regional Reference Centre for Inherited Bleeding Disorders, University Hospital of Parma, I 43100 Parma, Italy; atagliaferri@ao.pr.it (A.T.); acoppoladoc@gmail.com (A.C.); 6 Department of Oncology, Centre for Bleeding Disorders and Coagulation, Careggi University Hospital, I 50139 Florence, Italy; giancarlo.castaman@unifi.it (G.C.); linaris@aou-careggi.toscana.it (S.L.); 7 Regional Reference Centre for Inherited Bleeding and Thrombotic Disorders, Transfusion Medicine, “Regina Margherita” Children Hospital, I 10100 Turin, Italy; pollio.berardino@gmail.com; 8 Department of Hematology, University Hospital Policlinico “Umberto I”, I 00100 Rome, Italy; santoro@bce.uniroma1.it; 9 Regional Reference Centre for Hemorrhagic Diseases, IRCCS Istituto “Giannina Gaslini”, I 16100 Genoa, Italy; laurabanov@gaslini.org; 10 Hematology Unit, Thrombosis and Hemostasis Reference Regional Centre, University of Palermo, I 90100 Palermo, Italy; mariasanta.napolitano@unipa.it; 11 Department of Internal Medicine and Therapeutics, University of Pavia, I 27100 Pavia, Italy; p.preti@smatteo.pv.it; 12 Regional Reference Centre for Hemophilia and Coagulation Diseases, Azienda Ospedaliera Pugliese “Ciaccio”, I 88100 Catanzaro, Italy; ritacarlottasantoro@gmail.com; 13 SOBI, SE-11276 Stockholm, Sweden; e_santagostino@hotmail.com; 14 Department of Life Sciences and Biotechnology, Section of Biochemistry and Molecular Biology, University of Ferrara, I 44121 Ferrara, Italy; francesco.bernardi@unife.it

**Keywords:** rFVIIIFc, Hemophilia A, pharmacokinetics, one compartment model, two compartment model, non-compartmental analysis, Efmoroctocog alfa

## Abstract

**Background/Objectives:** A compartmental pharmacokinetics (PK) analysis of new extended half-life FVIII concentrates has never been performed in a large cohort of hemophilia patients. An improved PK analysis of individual outcomes may help to tailor hemophilia replacement treatment. **Methods:** PK outcomes after the infusion of a standard single dose of Efmoroctocog alfa were collected from 173 patients with severe/moderately severe hemophilia A in 11 Italian hemophilia centers. Factor VIII clotting activity (FVIII:C) was measured by one-stage clotting assay (OSA) in all patients, and chromogenic substrate assay (CSA) in a subgroup (*n* = 52). Fifty patients underwent a comparative PK assessment with standard half-life (SHL) recombinant FVIII (rFVIII) products. Non-compartmental analysis (NCA), one compartment model (OCM), and TCM were used to analyze the decay curves of all patients, and one-way paired ANOVA to compare the PK outcomes. **Results:** All 173 PKs conformed to the NCA and OCM, but only 106 (61%) conformed to the TCM based on the biphasic features of their decay curves. According to the TCM, the Beta HL and MRT of rFVIIIFc were 20.42 ± 7.73 and 25.64 ± 7.61 h, respectively. ANOVA analysis of the outcomes from the three PK models showed significant differences in clearance, half-life (HL), and mean residence time (MRT) (*p* < 0.001 for all parameters). As anticipated, the HL and MRT of rFVIIIFc were longer than those of SHL rFVIII. Comparing OSA with CSA outcomes, Cmax resulted higher when measured by CSA (*p* = 0.05) and, according to TCM, Beta HL resulted longer when measured by OSA (*p* = 0.03). FVIII:C trough levels obtained with SHL concentrates were significantly lower than those obtained with rFVIIIFc at each post-infusion time point. **Conclusions:** In a large group of hemophilia A (HA) patients, three different PK models confirmed the improved pharmacokinetic (PK) characteristics of rFVIIIFc, compared with standard half-life rFVIII concentrates. The TCM only fits two-thirds of the PKs, highlighting their biphasic decay and a long Beta half-life. In these patients, the TCM would be preferable to properly evaluate individual PK features.

## 1. Introduction

Hemophilia A is the most frequent inherited bleeding disorder, due to the deficiency of procoagulant FVIII (FVIII:C). The disease is classified according to the FVIII:C level as follows: severe < 1 IU/dL, moderate 1–5 IU/dL, and mild 6–40 IU/dL. Being the gene regulating the FVIII:C synthesis located on X chromosome, only the males (XY) are affected. The women (XX) are the carriers of the disease, apart those with skewed inactivation of the wild-type X-chromosome.The deficiency of FVIII:C causes a failure in the thrombin generation and, consequently, a decrease in fibrin clot formation. The bleedings affect the joints and skeletal muscles and are responsible for chronic arthropathy. The prevalence of severe hemophilia in Italy and other developed countries is 17.1/100,000 males for all hemophilia A and 6.0/100,000 males for severe hemophilia A [1]. Since the discovery that hemophilia was due to a deficiency of a factor of the blood [2], the first treatment involved the transfusion of blood or fresh frozen plasma from unaffected people [3] and soon after, of the cryopreciptate [4], produced by the rapid freezing and thawing of about 200 mL of plasma. The high volume (10–20 mL) and low factor VIII specific activity of cryoprecipitate (2–3 IU/mL) prompted the production of lyophiliazed plasma derived factor VIII (pdFVIII) concentrates of intermediate [5] and high purity from the large pool of plasma [6]. Unfortunately, the blood born viruses, HIV and HCV, present in these products, contaminated and killed about 80% of the treated hemophilia patients [7,8]. The pharmaceutical industry implemented the production of plasma-derived clotting factor concentrates with virucidal methods, and no new plasma-derived infection was reported after June 1985 [9]. The issue of viral contamination of the clotting factor concentrates was fixed after the introduction of recombinant FVIII concentrates, initially standard half-life (SHL rFVIII), with a half-life of about 12 h, and afterward extended half-life (EHL rFVIII), with a half life of about 18 h [10]. Unfortunately, the worst side effects of replacement therapy, i.e., the development of antibodies neutralizing the activity of infused FVIII, so far affects 26.8% and 44.5% of hemophilia A patients treated with pdFVIII or rFVIII concentrates, respectively [11]. Notwithstanding, the IV replacement therapy with FVIII concentrates, given on demand or by regular prophylaxis, avoided most of the bleeding and improved the quality of life of about 70% of hemophilia A patients. The approach to prophylaxis has become easier, safer and more effective during the last 10 years by implementing no-replacement therapy, like Emicizumab [12] and Concizumab [13], a new generation of monoclonal antibodies, permitting thrombin generation even in the presence of anti-FVIII antibodies. The large interpatient variability of factor VIII (FVIII) PK has been reported for several FVIII concentrates, while intra-patient variability is less significant. PK is mandatory for all new FVIII products during the phase I/II of clinical development programs. According to the recommendations of the International Society of Thrombosis and Hemostasis (ISTH), the PK of new clotting extended half-life (EHL) recombinant FVIII (rFVIII) concentrates should be evaluated in comparative studies against standard half-life (SHL) rFVIII concentrates, in small-medium size populations of 20–40 severe hemophilia A (HA) patients [14,15]. Unfortunately, the source data of PKs of newly developed clotting factor concentrates, carried out for regulatory purposes, are not always publicly available.

Efmoroctocog alfa (rFVIIIFc) was Italy’s first licensed EHL rFVIII product. To date, its PK performance has been described in three studies: (i) one pre-licensure study on 15 hemophilia A patients where only the OCM was used [16], (ii) in one single-center study on 18 patients where NCA was adopted [17], and (iii) one multicenter study including 114 patients where NCA was used [18]. Of note, TCM was never considered as a model to investigate the PK of rFVIIIFc, probably because it is too selective and reduces the PK studies’ size. With this background, AICE developed practical recommendations for performing PK assessments, according to the guidelines of the FVIII/FIX and Rare Bleeding Disorders ISTH-Standardization Sub-Committee (ISTH-SSC) [14,15]. AICE provided its members with a free-of-charge service for PK analysis using WinNonlin 7.0 (Certara, Radnor, PA, USA) during therapeutic switches, from SHL-rFVIII/IX to EHL-rFVIII/IX concentrates.

rFVIIIFc was the first EHL-rFVIII available in Italy; therefore, a vast amount of data were collected from patients switching from SHL-rFVIII to rFVIIIFc who underwent PK studies with the new concentrate, in some cases also in comparison with the previous concentrate. All the PK studies were analyzed using three PK methods (NCA, OCM, and TCM). Thanks to the availability of this large amount of data, a retrospective multicenter study was designed to perform a PK comparative analysis through NCA, OCM, and TCM, and better define the PK features of rFVIIIFc.

## 2. Subjects and Methods

Authorization: The study was approved as “observational” by the Ethical Committee of the University of Perugia and by the other University Hospitals on 26 October 2022, and 22 November 2022, respectively. Afterward, each Ethical Committee of the participating HTCs approved the study. Moreover, AICE supported the participating HTCs to submit the study protocol to local Ethical Committees. 

Inclusion/exclusion criteria: The main inclusion criterium was the ongoing treatment with Efmoroctocog alfa. The patients have been switched from another rFVIII concentrate some months before enrollment in this study. The presence of anti-FVIII:C antibodies, at enrollment or even in the past, was the major exclusion criterion. All patients signed the the informed consent form, approved by the Ethical Committee of each participating hemophilia center. 

Patients included: Severe/moderately severe (i.e., FVIII < 2%) HA patients have been enrolled in the PK study. All patients signed an informed consent to undergo the PK study.

FVIII:C assay: Factor VIII:C assay has been performed in the coagulation laboratories of each participating hemophilia centers by means of the One-Stage Activated Partial Thromplastin Time (APTT), using FVIII immune-depleted plasma as the substrate, and activated phospholipids, Imidazole buffer and calcium chloride as reagents. The reference calibration curve has been constructed by an APTT of 6 dilutions (from 1/10 to 1/360) of an international reference FVIII plasma standard. Three dilutions (1/10, 1/20 and 1/40) of each patient’s citrated plasma sample have been compared to the reference curve, and the FVIII:C has been calculated using the parallel lines method. The FVIII:C assay has also been performed using the chromogenic method in the laboratories of two hemophilia centers. In the first step, thrombin activates FVIII, turning it to to FVIIIa, which converts bovine FX to activated FXa, together with activated bovine FIX (FIXa), phospholipids (PL) and calcium chloride. Afterwards, the concentration of FXa was measured by the amount of hydrolyzed a p-nitroaniline. Both the procedures and the range of normal values of FVIII:C (60–150 IU/dL) have been reported in detail in a previous paper of ours [19]. 

### 2.1. Blood Collection and PK Design

The PK of rFVIIIFc was assessed at the time of the therapeutic switch from SHL-rFVIII concentrates to guide clinicians in tailoring the treatment, according to the patient’s individual needs and PK. rFVIIIFc single-dose PK was performed in 173 HA patients, along with a comparative study with the previous SHL-rFVIII in a subgroup (*n* = 51). Clinical and laboratory data were collected through a specific case report form (CRF) provided to clinicians. The CRF included: patient ID, age, ABO blood group, von Willebrand factor antigen (VWF:Ag), body weight, name and dose of rFVIII infused, FVIII:C concentration at baseline and at each post-infusion time. Patients with inhibitor history were excluded, as were those for whom PK assessment was performed without a minimum washout period of at least 96 h from the previous FVIII infusion. PK assessments were performed at each HTC according to the local clinical practice and AICE recommendations. The AICE protocol for PK assessment required: (1) a minimum washout period of 96 h from the last FVIII injection; (2) an intravenous infusion of a 40–50 IU/Kg dose of either SHL-rFVIII or EHL-rFVIII products over 2–3 min; (3) FVIII:C assay before and after 0.25, 6 or 9, 24, 48, 72, and 96 h post-infusion of Elocta and up to 48, or 72, or 96 h for SHL rFVIII concentrate. FVIII:C had been measured locally at each HTC by OSA, according to the local standard methods, and by CHA, if available. An unrestricted grant from AICE supported the study’s accomplishment. 

### 2.2. PK Methods and Statistical Analysis

PK parameters were assessed through 3 different models, NCA, OCM, and TCM. The OCM and TCM outcomes have been submitted to diagnostic procedures to check the goodness of curves’ fitting on maximum likelihood, according to the following parameters: Corr (Obs/Pred) outcomes, Sum of Squared Residuals (SSR) [20], Akaike Information Criterion (AIC) [21] and Schwarz Information Criterion (SIC) [22].

Each PK method provided the following parameters: 1-NCA: area under the curve (AUC), % of extrapolated AUC, moment of AUC (AUMC), C max, in vivo recovery (IVR), mean residence time (MRT), terminal Lambda_z half-life, clearance (Cl), and volume of distribution at the steady state (Vss). 2-OCM: AUC, AUMC, Cmax, MRT, the elimination HL from the central compartment (K1_0 HL), Cl, and Vss 3-TCM: AUC, AUMC, Cmax, MRT, K1_0, Cl, Vss, retrograde clearance (CLD2) from the extra-vascular to vascular compartment, Alpha HL (h) and Beta HL (h), the first and the second parts of the decay curve.

Overall, 173 patients with severe (*n* = 131) or moderately severe (*n* = 42) HA from 11 Italian HTCs underwent a single-dose PK with rFVIIIFc. FVIII:C was measured by OSA in all samples of each PK and by CSA in a subgroup of 52 patients (30%). A comparative PK assessment with previous SHL-rFVIII was performed in 50 patients (29%). Samples up to 96 h after the infusion of rFVIIIFc were always collected. The blood sampling after the infusion of SHL-rFVIII products was prolonged up to 48, 72, and 96 h in 15 (30%), 24 (48%), and 11 (22%) patients, respectively.

AICE provided a free-of-charge PK service for all HTCs using Phoenix WinNonlin 7.0. because the assessment of PK parameters was not available at each Italian HTC. The paired PK parameters of rFVIIIFc obtained through the 3 different methods, NCA, OCM, and TCM, have been compared by a one-way ANOVA. The outcomes of paired PKs, SHL-rFVIII vs. rFVIIIFc, were compared by paired t Student’s test.

## 3. Results

### 3.1. PK Parameters Obtained by NCA, OCM, and TCM

All 173 PKs performed with rFVIIIFc fit the NCA and OCM, but only 61% (*n* = 106) fit the TCM (Table 1a). According to NCA, the PK assessment schedule was good, since the extrapolated to infinity and lost AUC was only 3.23 ± 2.1% (mean ± 1 SD). The mean value of rFVIIIFc IVR by NCA was 2.22 ± 0.58 IU/dL/IU/kg, with a Cmax of 94.46 ± 32.65 IU/dL. 

**Figure 1:**
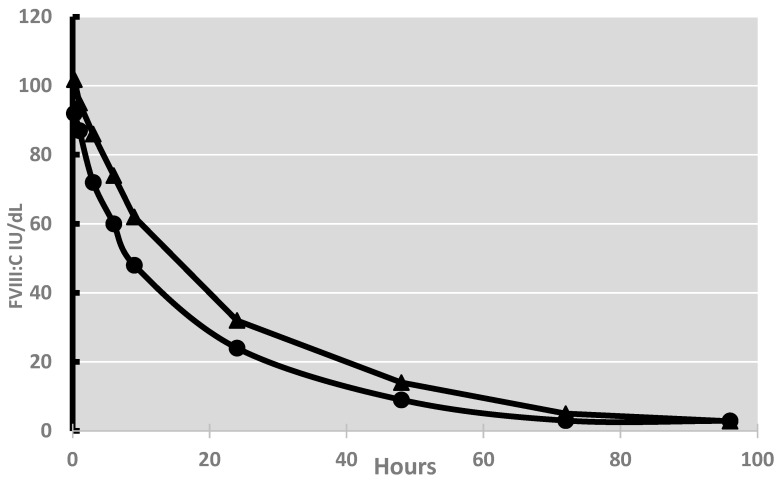
Decay of FVIII:C of Efmoroctocog alfa in hemophilia patients of group A (triangles) and B (circles).

We split the PK parameters obtained by NCA into two groups to infer why TCM did not model 67 PKs: group A (*n* = 106), constituted of PKs fitting the TCM, and group B (*n* = 67), those who did not. Table 1b shows the PK parameters obtained by NCA in groups A and B, and Figure 1 reports the FVIII:C decays of both groups. According to NCA, the mean Cmax was lower (*p* = 0.05), and the mean Lambda_z HL was longer (*p* =0.05) in those PKs, which also fit the TMC model (group A). This indicates that the shape of the decay of the PK curves of group A was biphasic (flatter terminal phase) and that of group B was steeper, monophasic, and for this reason, excluded by TCM. According to TCM, the retrograde clearance from the extravascular space to the plasma compartment (CLD2) was faster than the Cl (CLD2, 4.82 ± 1.78 vs. Cl, 2.50 ± 1.07 mL/h/kg), greatly impacting the slope of the terminal part of the curve and making the decay of FVIII:C biphasic in approximately two-thirds of patients.

PK outcomes of rFVIIIFc obtained in the 106 patients fitting NCA, OCM, and TCM were compared by one-way ANOVA analysis (Table 2). AUC and Cmax resulted similar across the three PK methods, while AUMC calculated by TCM was significantly larger than by NCA and OCM (*p* = 0.006). Cl calculated by OCM resulted faster than Cl by NCA and TCM (*p* = 0.008). The Vss by NCA resulted larger than that by OCM and TCM (*p* = 0.003). MRT by TCM resulted longer as compared to MRT by NCA and OCM (*p* = 0.0003). Beta HL was significantly longer than Lambda_z HL and K 1-0 HL (*p* = 0.0001). The paired comparison of diagnostics (Corr (Obs/Pred), AIC, SBC, and SSR) between the outcomes obtained with OCM and TCM using the paired *t*-test showed a statistically better performance of TCM (Supplementary Table S1). These observations imply that in these patients (*n* = 106) the TCM would be preferable to properly evaluate individual PK features.

### 3.2. OSA vs. CSA

FVIII:C was measured by both OSA and CSA (according to the local laboratory standard procedures) on blood samples obtained during PK studies performed on 52 patients, in two HTCs. PK parameters obtained in all 52 patients fit the NCA and OCM, while only those obtained in 34 patients fit the TCM (Table 3). According to NCA and OCM, Cmax resulted higher when measured by CSA vs. OSA (*p* = 0.05). According to TCM, Beta HL resulted longer when measured by OSA (*p* = 0.03), The paired comparison of diagnostics between the outcomes of OSA and CSA, according to OCM and TCM, is reported in Supplementary Table S2.

### 3.3. SHL-rFVIII vs. rFVIIIFc PK Analysis

For this comparative analysis, all PK studies (*n* = 50) were analyzed by NCA and OCM, whereas the data of only 32 patients fit the TCM (Table 4). Cmax and IVR of SHL-rFVIII and rFVIIIFc were similar. AUC and AUMC of rFVIIIFc were larger than those of SHL-rFVIII, due to different FVIII:C time sampling designs and its longer decay. Alpha HL was similar, while the Beta and Lambda_z HL of rFVIIIFc, compared to SHL-rFVIII, were 2 and 3 h longer, respectively. MRT of rFVIIIFc was consistently longer than that of SHL-rFVIII, regardless of the model used; NCA, OCM, or TCM (*p* = 0.001, *p* = 0.004, and *p* = 0.05, respectively, by paired t Student test). SHL-rFVIII clearance resulted faster than that of rFVIIIFc by NCA, OCM, and TCM (*p* = 0.04 for all), and the Vss was larger by NCA and TCM (*p* = 0.05 for both). The paired comparison of diagnostics between outcomes of rFVIIIFc vs. SHL-rFVIII is reported in Supplementary Table S3. Table 5 shows FVIII:C levels measured at 48, 72, and 96 h after SHL-rFVIII and rFVIIIFc infusions. FVIII:C levels obtained after rFVIIIFc resulted consistently higher than those obtained with SHL-rFVIII, *p* = 0.003, *p* = 0.004, and *p* = 0.05 at 48, 72 and 96 h, respectively.

### 3.4. Analysis According to Blood Groups

By comparing patients with O (*n* = 80) versus non-O (*n* = 93) blood group, VWF:Ag levels resulted significantly lower (85.8 ± 21.1 IU/dL vs. 125.3 ± 44.5 IU/dL; *p* = 0.004), Lambda-z HL shorter (16.8 ± 3.5 vs. 19.9 ± 3.6 h; *p* = 0.05), and Beta HL shorter (17.5 ± 4.4 vs. 22.5 ± 6.5 h; *p* = 0.01) in the former group.

## 4. Discussion

About 40 years ago, NCA was used for the first time to investigate the pharmacokinetics of clotting factor concentrates [23]. Later, the ISTH subcommittee [14] defined the rules of pharmacokinetics of new clotting factor concentrates. As reported by Gabrielsson J and Weiner D [24,25], the followers of NCA can be compared to people collecting dry flowers, and those using the compartmental models are likely people who prefer to grow and gather fresh flowers. All available PK methods to calculate rFVIIIFc PK parameters have been used for the first time in a large hemophilia A population. The log/linear best fitting of FVIII:C/time data by NCA allowed to analyze all the decay curves, independently from their shape. On the contrary, the FVIII:C/time linear/linear fitting of the compartmental models allows using all PK points, but the decay curves must be consistent with each PK method. The Corr (Obs/Pred) according to OCM and TCM is a good estimate of each method error (R), i.e., the difference between the observed and predicted values. All data of PKs fitted NCA and OCM but only 61.3% of them fit the TCM. In other words, 38.7% of PKs did not fit the TCM because of their monophasic and steeper decay, i.e., a higher Cmax and shorter HL. These are novel findings obtained by comparing the EHL PK profiles by multiple approaches for the first time. The underlying biological components warrant further investigation. According to TCM, the decay of 61.3% rFVIIIFc PKs was biphasic, with a long Beta HL of 20.42 ± 7.73 h and MRT 25 ± 7.61 h. The large inter-patient variability of PK outcomes may suggest the presence of environmental/genetic components modulating the PK features of EHL FVIII concentrates in a noticeable proportion of HA patients.

The PK analysis also differed by the number of FVIII:C/times points used: regarding the 173 rFVIIIFc PKs, all continued up to 96 h, and the lost AUC was 3.23%. On the contrary, the corresponding loss of AUC regarding the decay curves of SHL rFVIII concentrates, stopped at 48, 72, and 96 h, was 5.40%, 4.55%, and 3.15%, respectively.

Furthermore, according to all diagnostics, the TCM fits the decay of 61.3% of curves better than OCM, as shown by its lower SSR and better Corr(Obs/Pred). The FVIII IVR of rFVIIIFc by OSA was quite good, 2.12 ± 0.52 dL/kg, being the Cmax 101.05 ± 25.65 IU/dL, like the mean value (2.20 dL/kg) reported in the rFVIIIFc PK multicenter study conducted in France [18].

As expected, the IVR by CSA was slightly higher, 2.44 ± 0.61 dL/kg, according to the Cmax 116.36 ± 29.35. Similar discrepancies between IVR by OSA and CSA have also been observed for BDD rFVIII [19,26], N8-GP [27], and rFVIII full-length concentrates [28]. On the contrary, the MRT and Lambda_z HL, according to FVIII:C by CSA, proved to be shorter than the corresponding values by OSA, which means that the decay curves of FVIII:C by CSA are steeper than those by OSA.

MRT by TCM resulted significantly longer than by NCA or OCM (*p* = 0.0003).

No highly significant differences among AUCs by NCA, OCM, and TCM were observed (*p* = 0.09), but the AUMC by TCM resulted larger than that by NCA or OCM (*p* = 0.006). The Cmax of TCM, corresponding to the extrapolated concentration of FVIII at time 0 during the Alpha phase, is higher than the Cmax of NCA and OCM because the decay curves of TCM are significantly biphasic, even though the difference did not reach the level of statistical significance (*p* = 0.09). On the contrary, Lambda_z HL was shorter than Beta HL, being the first derived only from the best aligned FVIII/time points on a log/linear plot, and the second from the last, generally flatter, part of the decay linear/linear curve.

The source data FVIII:C/time fit better using the TCM than the OCM, as shown by the lower SSR and SSR/n of TCM, even though it was derived from the lower number of PKs best fitting this model, 106 out of 173, i.e., 61.3% of total PKs. On the contrary, about the same values of the drug flow rate out from the central (plasma) compartment, K 1-0 HL, have been observed as far as OCM and TCM were concerned.

The retrograde clearance (CLD2) from the extravascular to central compartment, higher than Cl, determined the biphasic decay and the long Beta HL of rFVIIIFc. Clearance from plasma compartment and Vss by NCA, OCM, and TCM resulted quite similar. They were not determined by the shape of the decay curve but only by the dose administered and AUC.

NCA, since 1985 [23], and recently [18], has been preferred over compartmental models when analyzing the PK data of rFVIIIFc, because it is a less demanding and more robust PK method, working on the log-transformed concentration/time data. Unfortunately, NCA does not provide the diagnostics for the best-fitting drug’s concentration/time points. The advantage of NCA and OCM is that both methods can evaluate all the decay curves, while the TCM accepts only those that best fit the model. OCM was used to analyze the first few PKs of rFVIIIFc of the phase 1 regulatory study in 15 patients [16]. The compared analysis, by one-way ANOVA of the 106 PKs fitting the TCM with the corresponding PKs fitting the OCM and analyzed by NCA, showed large and statistically significant differences among the Half-lives and MRTs derived by the three different methods, the best always the performances of TCM. The results of the PKs best fitting the TCM show how many patients can receive the best outcome from the rFVIIIFc concentrate. Both Lambda_z_HL and Beta HL resulted longer in patients with non-0 blood group and higher VWF levels, due to the faster clearance of VWF/FVIII complex by anti-A and anti-B agglutinins of blood group 0 [29,30,31].

## 5. Conclusions

As a robust method, the NCA could evaluate all the PK curves independently from their monophasic or biphasic decay. The sample timing of the study was quite good because, according to NCA, only about 6% of the AUC went lost in the worst PKs. The data of rFVIIIFc PKs fit the TCM better than OCM in 59.3% of patients, as shown by the paired comparative analysis. TCM highlighted the biphasic decay of the 106 curves that best fit this model, and potentially a noticeable proportion of HA patients with modulated and better PK features: Beta HL was significantly longer than Lambda_z HL or K 1-0 HL. Also, MRT by TCM was considerably more prolonged than by NCA or OCM according to the paired comparison by ANOVA. The comparative study of SHL rFVIII vs. rFVIIIFc displayed a significant difference in the trough levels at 48 and 72 h, and still substantial at 96 h.

This first report of the ClinPhaGenE multicenter study shows a large inter-patient variability of PK outcomes. Apart from the well-known variability of the FVIII:C assay and the differences between the NCA, OCM, and TCM, we hope, in the frame of the ClinPhaGenE study, to be able to evaluate the *F8* and extra *F8* genotypes as modifiers of the pharmacokinetics of rFVIIIFc, as we did for other FVIII concentrates [32].

## Figures and Tables

**Table 1:** (a) The outcomes of Efmoroctocog alfa PKs by non-compartmental analysis (nca), one-compartment model (ocm), and two-compartment model (tcm). (b) The outcomes of Efmoroctocog alfa PKs by NCA (*n* = 173) have been split into group A, those analyzed also by TCM (*n* = 106), and group B, those analyzed only by NCA (*n* = 67). The mean Cmax of group A is about 9 IU/dL lower than that of group B, while the mean Lambda_z HL is about 3 h longer. This means that the decay of group B (higher Cmax and shorter HL) is steeper, i.e., monophasic, and that of group A (lower Cmax and longer HL) is flatter, i.e., biphasic.

**(a)**												
			**AUC**	**AUMC**	**Cmax**	**IVR**	**MRT**	**Lambda_z HL**	**K1_0 HL**	**Cl**	**Vss**	**AUC_%Extrap**
	**N**		**Uxh/dL**	**Uxh^2^/dL**	**IU/dL**	**IU/dL/IU/kg**	**h**	**h**	**h**	**mL/h/kg**	**mL/kg**	**%**
NCA	173	Mean	1978	39,793	94.46	2.22	19.09	17.27		2.36	55.08	3.23
		1 SD	790	23,400	32.65	0.58	4.78	5.85		0.95	34.41	3.00
OCM	173	Mean	1817	40,811	92.11		20.16		13.98	2.76	49.04	
		1SD	819	31,764	30.59		7.15		4.95	1.28	12.77	
TCM						Alpha HL (h)		Beta_HL (h)				CLD2 (mL/h/kg)
	106	Mean	1870	51,790	99.74	3.74	25.64	20.42	13.72	2.50	59.32	4.82
		1SD	758	38,647	41.01	3.00	7.61	7.73	4.68	1.07	18.93	1.78
**(b)**												
				**Non-Compartmental Analysis**								
						**AUC**	**AUMC**	**Cmax**	**MRT**	**Lambda_z HL**	**Cl**	**Vss**
**Groups**				**N**		**Uxh/dL**	**Uxh^2^/dL**	**IU/dL**	**h**	**h**	**mL/h/kg**	**mL/kg**
A				106	Mean	1884	39,461	92.88	19.94	18.87	2.36	61.08
					1 SD	699	21,740	32.77	4.43	5.88	0.99	27.65
B				67	Mean	2072	40,616	101.65	18.11	15.88	2.41	52.30
					1 SD	907	26,696	38.42	5.32	6.51	0.96	26.01

**Table 2:** One-way ANOVA analysis of PK parameters of Efmoroctocog alfa obtained from 106 PK studies fitting NCA, OCM, and TCM.

	Groups	N	Mean	Variance	Variance between Groups	F	*p*
AUC (Uxh/dL)	NCA	106	1884	488,274	2,509,245	2.40	0.09
	OCM	106	1689	502,411			
	TCM	106	1870	574,613			
AUMC (Uxh^2^/dL)	NCA	106	39,461	472,632,451	13,903,028,009	7.67	0.006
	OCM	106	36,530	752,738,949			
	TCM	106	51,790	1,493,605,408			
Cmax (IU/dL)	NCA	106	92.88	1074	5922	2.44	0.09
	OCM	106	89.34	892			
	TCM	106	99.74	1681			
Clearance (mL/h/kg)	NCA	106	2.36	0.01	0.13	4.88	0.008
	OCM	106	2.84	0.02			
	TCM	106	2.50	0.01			
Vss (mL/kg)	NCA	106	61.08	7.64	92.90	10.60	0.003
	OCM	106	48.84	1.86			
	TCM	106	59.32	3.58			
HL (h)	Lambda_z HL	106	18.87	34.60	2804	34.50	0.0001
	K 1-0 HL	106	13.54	23.02			
	Beta HL	106	20.42	64.33			
MRT (h)	NCA	106	19.94	19.64	2467	29.51	0.0003
	OCM	106	19.54	47.91			
	TCM	106	25.64	57.83			

**Table 3:** The non-compartmental analysis (NCA), one-compartment model (OCM), and two-compartment model (TCM) outcomes of the study comparing the FVIII:C decay of Efmoroctocog alfa by one-stage assay (OSA) and chromogenic assay (CHA).

				AUC	AUMC	Cmax	IVR	MRT	Lambda_z HL	K1_0 HL	Cl	Vss	AUC_Extrap
		N		Uxh/dL	Uxh^2^/dL	IU/dL	IU/dL/IU/kg	h	h	h	mL/h^2^/kg	mL/kg	%
NCA	OSA	52	Mean	2149	44,699	101.05	2.12	19.46	17.24		2.51	58.01	3.29
			1 SD	851	27,388	25.65	0.52	4.54	6.36		1.05	26.13	2.80
	CHA	52	Mean	2265	43,162	116.36	2.44	18.09	16.98		2.37	54.96	4.92
			1 SD	889	26,148	29.36	0.61	4.48	7.95		1.04	31.19	3.21
OCM	OSA	52	Mean	2007	46,396	98.32		20.15		13.96	2.90	51.24	
			1 SD	943	39,457	24.18		7.12		4.93	1.32	12.46	
	CHA	52	Mean	2052	41,083	114.57		17.76		12.31	2.87	44.76	
			1 SD	946	33,013	28.52		6.18		4.28	1.50	42.92	
TCM							Alpha HL (h)		Beta HL (h)				CLD2 (mL/h^2^/kg)
	OSA	34	Mean	2169	61,128	123.24	3.01	25.43	19.23	13.19	2.60	59.98	5.42
			1 SD	988	48,174	63.13	2.31	7.92	7.06	5.32	1.21	20.64	1.50
	CHA	34	Mean	2226	59,307	120.29	4.23	24.24	24.26	12.60	2.53	56.13	5.90
			1 SD	1041	45,966	31.17	3.68	7.28	8.30	3.93	1.30	21.31	1.70

**Table 4:** The outcomes of non-compartmental analysis (NCA), one-compartment model (OCM), and two-compartment model (TCM) of the PK study comparing the SHL rFVIII concentrates vs. Efmoroctocog alfa.

				AUC	AUMC	Cmax	In Vivo Recovery	MRT	Lambda_z HL	K10_HL	Cl	Vss
		N		Uxh/dL	Uxh^2^/dL	IU/Dl	IU/dL/IU/kg	h	h	h	mL/h/kg	mL/kg
NCA	SHL rFVIII	50	Mean	1320	20,877	76.84	2.33	15.09	17.33		2.83	63.82
			1 SD	669	14,414	27.43	0.69	4.73	11.46		1.17	36.55
	Elocta	50	Mean	1808	39,134	76.21	2.19	20.85	20.20		2.03	56.22
			1 SD	630	21,129	21.32	0.48	4.78	9.30		0.58	25.91
OCM	SHL rFVIII	50	Mean	1159	19,231	76.36		15.17		10.52	3.44	47.72
			1 SD	596	15,848	27.06		4.56		3.16	1.80	15.52
	Elocta	50	Mean	1710	41,685	76.68		22.79		15.80	2.31	49.18
			1 SD	641	25,664	21.79		6.71		4.65	0.76	12.09
							Alpha HL (h)		Beta HL (h)			
TCM	SHL rFVIII	32	Mean	1287	34,292	75.90	3.78	24.16	20.98	11.77	6.35	141.21
			1 SD	572	26,928	23.28	2.21	9.90	10.04	3.36	1.82	33.91
	Elocta	32	Mean	1846	63,636	79.80	3.59	29.55	22.6	15.97	4.63	126.13
			1 SD	882	79,504	21.13	2.49	11.98	12.78	4.55	1.31	17.50

**Table 5:** FVIII:C troughs at 48, 72, and 96 h after SHL-rFVIII and Efmoroctocog alfa infusions.

		N	FVIII:C IU/dL (Mean + 1 SD)	Student’s *t*-Paired Test
Trough at 48 h	SHL rFVIII FVIII	15	2.16 ± 1.77	*p* = 0.0003
	Efmoroctocog alfa	15	7.87 ± 3.57	
Trough at 72 h	SHL rFVIII FVIII	24	2.78 ± 1.01	*p* = 0.004
	Efmoroctocog alfa	24	4.99 ± 3.24	
Trough at 96 h	SHL rFVIII FVIII	11	2.35 ± 1.10	*p* = 0.05
	Efmoroctocog alfa	11	4.01 ± 2.18	

## Data Availability

All data are available at request to Massimo Morfini, MD.

## Supplementary Materials

The following supporting information can be downloaded at: https://www.mdpi.com/article/10.3390/jcm13174986/s1, Table S1: The paired comparison between the One-Compartment Model (OCM) and Two-Compartment Model (TCM) fitting parameters of Efmoroctocog alfa PKs shows the better performance of TCM: lower AIC, SBC, and, particularly, SSR. The Corr(Obs/Pred) values by TCM are between 0.9999 and 1.0 in about 33% of PKs; Table S2: The best fitting diagnostics of Efmoroctocog alfa PKs by the FVIII:C One Stage Assay (OSA) and Chromogenic Assay (CHA), ac-cording to the One-Compartment Model (OCM) and Two-Compartment Model (TCM); Table S3: The diagnostics of PKs of rSHL FVIII concentrate vs. Efmoroctocog alfa according to the One-Compartment Model and Two-Compartment Model.
