# Supplementary material for: Pharmacokinetics of Efmoroctocog alfa by Two-Compartment Model Highlights Hemophilia A Patients with Biphasic Decay, Long Mean Residence Time, and Beta Half-Life
Source: J Clin Med. 2024 Aug 23;13(17):4986. doi: 10.3390/jcm13174986 (PMC11396525; doi:10.3390/jcm13174986)
Supplement: Supplementary file 1 [file jcm-13-04986-s001.zip › jcm-3155295-supplementary.pdf]

		OCM (n=106)					TCM (n=106)		
	AIC	CORR_(Obs/Pred)	SBC	SSR		AIC	CORR_(Obs/Pred)	SBC	SSR
Mean	33.36	0.9932	33.26	411.51		18.05	0.9994	17.84	32.09
1SD	15.25	0.0090	15.51	189.36		14.93	0.0008	15.45	13.65

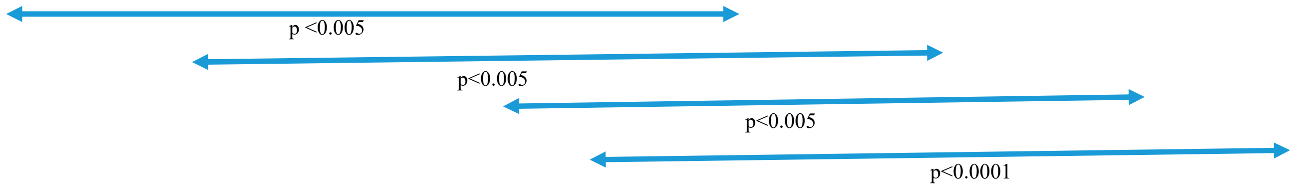

Supplementary Table S1 -The paired comparison between the One-Compartment Model (OCM) and Two-Compartment Model (TCM) fitting parameters of Efmoroctocog alfa PKs shows the better performance of TCM: lower AIC, SBC, and, particularly, SSR. The Corr(Obs/Pred) values by TCM are between 0.9999 and 1.0 in about 33% of PKs.

ONE COMPARTMENT MODEL												
		OSA							CHA			
**N**		**AIC**	**CORR_(OBS,PRED)**	**SBC**	**SSR**	**SSR/N**	**N**	**AIC**	**CORR_(OBS,PRED)**	**SBC**	SSR	SSR/N
52	Mean	35.97	0.9942	35.96	198.91	3.82	52	38.49	0.9926	38.47	328.63	6.32
	1SD	11.14	0.0067	11.32	138.35			10.46	0.0158	10.70	182.87	
TWO COMPARTMENT MODEL												
		OSA									CHA	
N	ID	AIC	CORR_(OBS,PRED)	SBC	SSR	SSR/N	N	AIC	CORR_(OBS,PRED)	SBC	SSR	SSR/N
34	Mean	23.23	0.9995	23.35	16.53	0.48	34	29.93	0.9983	30.01	61.24	1.80
	1SD	11.70	0.0005	12.00	13.30			16.13	0.0031	16.44	46.26	

Supplementary Table S2 - The best fitting diagnostics of Efmoroctocog alfa PKs by the FVIII:C One Stage Assay (OSA) and Chromogenic Assay (CHA), according to the One-Compartment Model (OCM) and Two-Compartment Model (TCM)

ONE-COMPARTMENT MODEL													
**SHL rFVIII**							**ELOCTA**						
**N**	**ID**	**AIC**	**CORR (OBS,PRED)**	**SBC**	**SSR**	**SSR/N**	**N**	**AIC**	CORR (OBS,PRED)	SBC	SSR	SSR/N
50	Mean	18.59	0.9974	18.01	20.82	0.59	50	22.24	0.9964	21.98	28.91	0.83
	1SD	6.37	0.0047	6.49	18.52			7.42	0.0058	7.42	26.36	

TWO-COMPARTMENT MODEL												
SHL rFVIII							ELOCTA					
N	ID	AIC	CORR_(OBS,PRED)	SBC	SSR	SSR/N	N	AIC	CORR_(OBS,PRED)	SBC	SSR	SSR/N
32	Mean	7.95	0.9997	7.08	2.94	0.12	32	17.98	0.9989	17.63	9.90	0.41
	1SD	10.28	0.0004	10.53	5.23			8.62	0.0025	8.63	17.17	

Supplementary Table S3- The diagnostics of PKs of rSHL FVIII concentrate vs Efmoroctocog alfa according to the One-Compartment Model and Two-Compartment Model
